# Evaluation of Thermal Decomposition Kinetics of Poly (Lactic Acid)/Ethylene Elastomer (EE) Blends

**DOI:** 10.3390/polym15214324

**Published:** 2023-11-04

**Authors:** Giordano P. Bernardes, Matheus P. Andrade, Matheus Poletto, Nathália R. Luiz, Ruth M. C. Santana, Maria M. de C. Forte

**Affiliations:** 1 Department of Mechatronic Engineering, Atlantic Technological University (ATU) Sligo, Ash Lane, F91 YW50 Sligo, Ireland; 2 Postgraduate Program in Engineering of Processes and Technologies (PGEPROTEC), University of Caxias Do Sul (UCS), Caxias Do Sul 95070-560, Brazil; mpandrade@ucs.br; 3 Laboratory of Polymeric Materials (LAPOL), School of Engineering, Federal University of Rio Grande do Sul (UFRGS), Porto Alegre 90010-150, Brazil; nathalia.rosa@ufrgs.br (N.R.L.); ruth.santana@ufrgs.br (R.M.C.S.); mmcforte@ufrgs.br (M.M.d.C.F.)

**Keywords:** PLA, polymer blends, compatibilization, thermal properties, solid-state reaction

## Abstract

The influences of ethylene-based elastomer (EE) and the compatibilizer agent ethylene-butyl acrylate-glycidyl methacrylate (EBAGMA) on the thermal degradation of PLA/EE blends were evaluated by the thermal degradation kinetics and thermodynamic parameters using thermogravimetry. The presence of EE and EBAGMA synergistically improved the PLA thermal stability. The temperature of 10% of mass loss (T_10%_) of PLA was around 365 °C, while in the compatibilized PLA/EE blend, this property increased to 370 °C. The PLA average activation energy ($\bar{E_{a}}$) reduced in the PLA/EE blend (from 96 kJ/mol to 78 kJ/mol), while the presence of EBAGMA in the PLA/EE blend increased the $\bar{E_{a}}$ due to a better blend compatibilization. The solid-state thermal degradation of the PLA and PLA/EE blends was classified as a D-type degradation mechanism. In general, the addition of EE increased the thermodynamic parameters when compared to PLA and the compatibilized blend due to the increase in the collision rate between the components over the thermal decomposition.

## 1. Introduction

Biodegradable polymers (BDPs) have been researched and developed as materials with the potential to replace conventional non-biodegradable polymers (n-BDPs). BDPs present the benefit of being extracted from diverse natural sources (e.g., cellulose and starch) [1] with low environmental impact and are divided into natural and synthetic polymers [2]. The natural BDP polysaccharides (such as chitosan, cellulose, starch), proteins (such as whey protein, corn zein, and soy protein) [3], and microbial polyesters (such as poly(β-hydroxyalcanoate) (PHA) and poly (3-hydroxybutyrate) (PHB) [4], as well as poly (hydroxybutyrate-*co*-hydroxyvalerate) (PHBV)) [5] are the most studied and employed. Synthetic polymers, such as polyesters, polyamides, polyurethanes, and polyureas [6], are biodegradable due to these chemical groups being susceptible to hydrolyzation. In terms of representants, poly (glycolic acid) (PGA) and poly (lactic acid) (PLA) [7], poly (butylene succinate) (PBS) [8], poly(ε-caprolactone) (PCL) [9], and poly (butylene adipate-*co*-terephthalate) (PBAT) [10] are the most important synthetic BDPs.

PLA is one of the most known and employed synthetic biodegradable polyesters, obtained from corn and potato starch [11] and commonly used in the biomedical area [12] and in additive manufacturing [13]. PLA is a brittle polymer at room temperature and shows glass transition temperature (Tg) around 60 °C [14]. In terms of mechanical behavior, PLA presents a higher elastic modulus than polypropylene (PP), acrylonitrile-butadiene-styrene (ABS), or polyamide (PA) [12]. Depending on the application, the low toughness and slow crystallization kinetics of PLA implies using plasticizers, nucleating agents, and flexible polymers (polyesters, conventional or thermoplastic elastomers) [15,16] to improve the balance of rigidity–toughness–processability. PLA blends with thermoplastic polyurethane (TPU), ethylene elastomers [17], polycarbonate (PC) [18], polyhydroxyalkanoate (PHA) [19], or PBAT [16], are commonly used for balancing the PLA rigidity–tenacity, allowing the production of materials with new properties and good reproducibility at low cost [20].

A polymer blend is affected by factors such as morphology, interfacial interaction between components, polymer viscosity, and particle size distribution (in the case of heterophasic mixtures). Some studies regarding PLA blends reported an improvement of the PLA mechanical properties in blends with ethylene-vinyl acetate (EVA) [21], poly (ethylene glycol) (PEG) [22], and PCL [23]. Since incompatible polymer blends present phase separation [24], properties can be improved using a compatibilizer agent (CA) [usually block or graft copolymers that can interact with both blend phases] [25]. Ethylene-acrylate copolymers, such as ethylene-glycidyl methacrylate (EGMA), ethylene-methyl acrylate-glycidyl methacrylate (EMAGMA) [16], and ethylene-butyl acrylate-glycidyl methacrylate (EBAGMA) [17], have been reported as good CAs for PLA blend compatibilization. The CA promotes a significant increase in the intermolecular interaction between the polymers’ domains, lowering the interfacial tension [26] and enhancing the mechanical properties [27]. The PLA/plasticized cellulose acetate (pAC) (85/15 *w*/*w*) blend compatibilized with AC-g-PLA [28] and PLA/bio polyethylene (bioPE) (80/20 *w*/*w*) blend compatibilized with EVA [29] have shown improved tenacity comparing with the respective blend without a compatibilizer agent.

Polymer blend components usually have different chemical structures and, thus, distinct thermal degradation behavior and kinetics mechanisms. Moreover, some degradation products may influence the other degradation processes and vice versa, changing the decomposition rate and the energy input to initiate the degradation process. The thermal degradation kinetics of polymers is usually assessed by isoconversional methods such as Flynn–Wall–Ozawa (FWO) [30], Kissinger–Akahira–Sunose (KAS) and Starink [31], as well as Friedman [30], and Advanced Isoconversional Model (AIC) [32]. The isoconversional methods allow the estimation of important parameters (such as activation energy) and are independent of reaction models [33]. Complementing the isoconversional evaluation, Criado’s method [31] evaluates reaction models and compares experimental data versus master curves to identify the most probable degradation mechanism.

Another important approach to assess a thermal degradation reaction is through the thermodynamics of the degradation reaction [34]. Thermodynamical parameters, molar changes in enthalpy (ΔH), entropy (ΔS), and free Gibbs energy (ΔG) and frequency factor by using activation (A) are used to evaluate the spontaneity of the degradation reactions and how these parameters are affected by the extension of conversion (α) and heating rate (β). Carrasco et al. [35] studied PLA/PA blend kinetics of thermal degradation, observing the increase of %PA in the blend which shifted PLA T_5%_ to higher temperatures and increased the apparent activation energy for thermal degradation. The authors observed that F2 (random degradation nucleation with two nuclei on the individual particle), R2 (phase boundary-controlled reaction), and D2 (2-dimension diffusion) reaction models are possible models of the kinetics of thermal degradation of PLA/PA 70/30 blend. Alhulaybi et al. [31] studied the thermal behavior of PLA through thermogravimetry, observing that PLA thermal degradation occurred in a single thermal event. The PLA kinetics of thermal degradation was assessed by isoconversional methods (Friedman, FWO, KAS, and Starink), and activation energy (E_a_) was estimated to be between 97–109 kJ/mol. PLA exhibited an R2-type (geometrical contraction model) reaction mechanism regardless of the heating rate based on Criado’s method.

Many papers discussing the PLA and PLA blend thermal degradation focus on their thermal stability at different atmospheres by TGA/DTG analysis. However, to the best of our comprehension, the literature offers a minor number of papers regarding a deeper discussion about how a compatibilizer agent can affect or modify the PLA thermal stability or degradation. In a previous work [36], we discussed the effect of the compatibilizer agent EBAGMA on the PLA/TPU (70/30% wt.) blend mechanical properties. The addition of 5 wt.% EBAGMA in the PLA/TPU blend increased the Izod impact absorption from 3.4 kJ/m^2^ to 5.0 kJ/m^2^ (while the PLA Izod impact was only 1.9 kJ/m^2^), evidencing an improved intermolecular interaction between the polymer molecules. This paper aims to investigate the effect of the compatibilizer agent EBAGMA on PLA/EE (ethylene elastomer) blends’ thermal degradation by evaluating the thermal decomposition kinetic and thermodynamic parameters using TGA/DTG analyses. The choice of thermoplastic elastomer EE was because of its non-polar nature (in contrast to PLA, a polar polymer), which requires a compatibilizer agent in a blend with PLA.

## 2. Materials and Methods

### 2.1. Materials

The main physical, rheological, and thermal data of the polymers, poly (lactic acid) (PLA) (NatureWorks, NE, USA) ethylene elastomer (EE) (DuPont, Brazil), and terpolymer ethylene-butyl acrylate-glycidyl methacrylate (EBAGMA) (DuPont, Brazil) are listed in Table 1.

### 2.2. Preparation of PLA/EE Blends

Table 2 shows the nomenclature and compositions (in wt.%) of the PLA/EE blends prepared without (PLA30EE) and with the compatibilizer agent (CA) EBAGMA (PLA30EE-C) as previously discussed [17]. The PLA blend components were simultaneously added into an internal mixer chamber model Haake Rheodrive 7 Rheomix OS (ThermoFisher, Waltham, MA, USA) (chamber temperature: 190 °C; rotors speed: 50 rpm; residence time 8 min). The polymers PLA, EE, and EBAGMA were dried at 30 °C for 24 h before processing to remove moisture.

### 2.3. PLA and PLA Blends Thermal Characterization

The thermal stability of PLA, EE, EBAGMA, and PLA blends was assessed in terms of mass loss (TG) and mass loss rate (DTG) through thermogravimetric analysis (TGA) using a thermogravimetric analyzer model Q50 calorimeter (TA Instruments, New Castle, DE, USA) in the N_2_ atmosphere. The tests were carried out from 25 to 600 °C at different heating rates (β) (5, 10, and 20 °C min^−1^) using approximately 10 mg of each sample. The temperature of 5 and 10% of mass loss (T_5%_ and T_10%_, respectively) and the DTG peak temperature (T_p_) were estimated for all samples in different β.

#### 2.3.1. Thermal Decomposition Kinetic Approach

The thermal decomposition kinetics of PLA, EE, EBAGMA, and PLA/EE blends were evaluated according to Flynn–Wall–Ozawa (FWO) (Equation (1)) and Vyazovkin (Equation (2)) models.

log (β) = log [A E_a_/g(α)] − 2.315 − 0.4567 (E_a_/RT) (1)

(2) $$g\left(\alpha \right)=\int_{0}^{\alpha }\frac{d\alpha }{f(\alpha )}=A\int_{0}^{t}exp(\frac{E_{\alpha }\left(\alpha \right)}{RT})dt=AJ[E_{\alpha }\left(\alpha \right),T]$$

where g(α), f(α), and A are, respectively, the integral form of the reaction model, the heating program, and the Arrhenius constant, and T(t) is also the heating program (assumed as linear).

The curves log β versus reciprocal of temperature (in absolute temperature) at different heating rates (β) were plotted, and the apparent activation energy (E_a_) (slope of the curve) at each extent of conversion (α) was determined for all samples. E_a_ (associated with the energy to the occurring event) and the frequency factor (A) (associated with the vibration frequency of the products from the reaction degradation, also known as Arrhenius parameters) were estimated using the isoconversional method, assuming the reaction rate at constant α is only affected by the temperature.

The Criado method [37] was used to determine and evaluate the degradation reaction mechanism in a solid-state reaction process for PLA, EE, EBAGMA, and PLA blends. This method uses a Z function, which depends on the conversion extent α (Z(α)) (Table 3). The comparison of the master curves (plotted from the theoretical values) with the experimental values indicates the most likely mechanism(s) of the solid-state reaction. The degradation mechanisms divide into nuclei formation (A_n_), phase boundary-controlled reaction (R_n_), diffusion (D_n_), random degradation nucleation (F_n_), and random chain scission (L_n_) processes [38].

#### 2.3.2. Thermodynamic Approach

The thermodynamic parameters of the degradation reaction [39] are functions of the extent of conversion (α) and are used in conjunction with the kinetics of thermal decomposition. The expressions for the thermodynamic parameters of the degradation frequency factor (A), molar changes in enthalpy (ΔH), entropy (ΔS), and Gibbs free energy (ΔG) are in Table 4. The heating rate for estimating T_p_ and E_a_ was 20 °C/min (estimated by the FWO method).

Where “β” is the heating rate (in K.min^−1^), “k_B_” is the Boltzmann constant (1.38 × 10^−23^ J K^−1^), “h” represents the Planck’s constant (6.67 × 10^−34^ J s), “T_p_” is the DTG peak temperature (in K), “T_α_” is the temperature (in K) at an extent of conversion α, and “R” is the gas constant (8.314 J mol^−1^ K^−1^).

## 3. Results and Discussion

### 3.1. Thermal Stability Evaluation in a Non-Oxidative Atmosphere

The poly (lactic acid) (PLA) is a polar semicrystalline polyester (–[HCCH_3_-CO-O]_n_–), the ethylene elastomer (EE) is a non-polar ethylene-α olefin copolymer (–[CH_2_-CH_2_]_x_-[CH_2_-CH(CH_2_(CH_2_)_m_CH_3_)]_y_–), and the compatibilizer agent EBAGMA is a terpolymer ethylene-butyl acrylate-glycidyl methacrylate (–[CH_2_-CH_2_]_x_-[CH_2_-CHCOOBu]_y_-[CH_2_-CCH_3_COOGly]_z_–) with non-polar and polar chain sequences. The polymer’s thermal stability is mainly related to the molecular weight and chemical and physical structure of molecules, affecting the type of intermolecular forces and the polymer’s thermal behavior.

**Figure 1:**
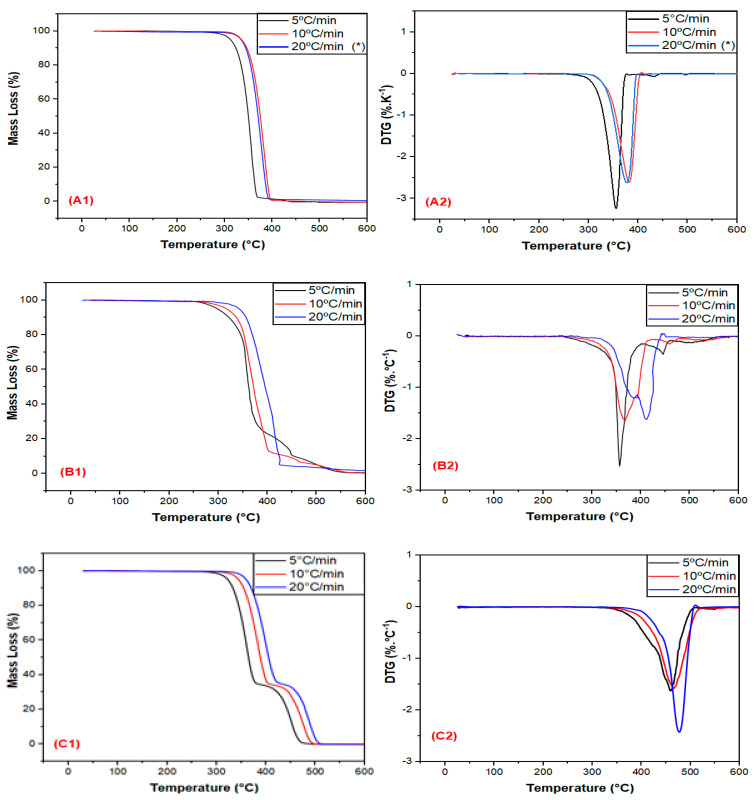
Mass loss and derivative curves vs. temperature of the PLA (**A1**,**A2**), EE (**B1**,**B2**), and EBAGMA (**C1**,**C2**) at different heating rates. * Originally published: [17].

Figure 1 shows the mass loss (TG) and derivative (DTG) curves of the PLA, EE, and EBAGMA as a function of temperature at different heating rates.

The thermal stability of PLA is affected by moisture, hydrolyzed monomers and oligomers, molecular weight, and residual metals [40], resulting in lower thermal stability than polyolefins [33]. The PLA thermal degradation occurs by chain-end scission with a hydroxyl group (–OH) and random scission at the main polymer chain [33], as well as unzipping depolymerization reactions [41]. These chain scission reactions divide into hydrolysis, oxidative degradation, cis-elimination, and inter/intramolecular transesterification reactions [41]. In terms of PLA degradation products, linear hydroxyl, ester, and carbonyl groups are the most important ones [42]. The PLA (Figure 1A) presented a single decomposition event regardless of the heating rate, as similarly observed by Ruz-Cruz et al. [43]. The increase in the heating rate shifted the PLA decomposition peak (T_p_) to higher temperatures due to thermal lag, heat transfer limitations, and the time-temperature superposition principle [44].

Table 5 shows the temperatures in which there is 5% (T_5%_) and 10% (T_10%_) of mass loss and the temperature (T_p_) where the degradation rate is maximum (or the derivative curve peak) of the PLA, EE, and EBAGMA as a function of temperature at different heating rates. The T_p,PLA_ occurred in the range 350–380 °C at different heating rates, in conformity with Karimpour-Motlagh et al. [44], Hayone et al. [45], and Abu Hassan et al.’s [46] findings. Based on T_5%_ and T_10%_ values at different heating rates, the PLA would be thermally stable and suitable for injection and extrusion processing. The ethylene elastomer EE, a non-polar elastomer mostly composed of ethylene monomers, has unsaturated butene as the main thermal degradation product and other vapors [17]. The EE (Figure 1B, Table 5) presented two decomposition events and no significant mass loss below 200 °C, indicating that this material is suitable for injection and extrusion processes as PLA. The T_p, EE_ occurred in the range 360–470 °C at different heating rates, as similarly reported for high-density polyethylene (HDPE) under a non-oxidative atmosphere [47,48,49]. In general, increasing the heating rate leads the polymer degradation to occur by two events in higher temperatures.

The acrylate compatibilizer EBAGMA is a random terpolymer composed of 66.75 wt.% ethylene, 28 wt.% n-butyl acrylate, and 5.25 wt.% glycidyl methacrylate. The bigger the n-butyl acrylate content, the better the engineering polymers’ thermal stability [50]. EBAGMA is the compatibilizer agent used in polymer blends with polyesters such as PET and PLA because of its high efficiency [51]. However, there is little data discussion in the literature on the decomposition mechanism of EBAGMA and yielded products. The different chemical structures in EBAGMA could undergo individual thermal decompositions and yield different products. However, EBAGMA presented a single decomposition event with T_p_ in the range 460–480 °C (Figure 1C, Table 5), which could be explained by the high ethylene content. Despite each domain undergoing individual thermal decomposition, the ethylene domain degradation controlled the overall reaction of EBAGMA, resulting in a similar thermal degradation exhibited by HDPE. The ethylene domain thermal degradation mechanism occurs by a random scission followed by a radical transfer process [52], and some possible yielding products are hydrocarbons, carboxylic acids, and aldehydes [53]. On the other hand, poly (n-butyl acrylate) thermal degradation occurs by random chain scissions [54], and some yielding products are butene, methacrylic acid, and anhydrides [55]. The GMA group in GMA-grafted polymers can undergo chemical reactions with polyester hydroxyl end-groups and generate graft copolymers at the interface, improving polymer blend compatibility [55,56,57,58]. Huang and Kang [56] evaluated the thermal stability of poly (glycidyl methacrylate) (PGMA) by TGA at different heating rates, observing that PGMA showed a single thermal decomposition event and an onset thermal decomposition around 280–300 °C. On the other hand, Lee et al. [57] reported that the poly (ethylene-co-glycidyl methacrylate) (EGMA) thermal decomposition, under a non-oxidative atmosphere, was a single-step event and the T_p_ was around 475 °C.

**Figure 2:**
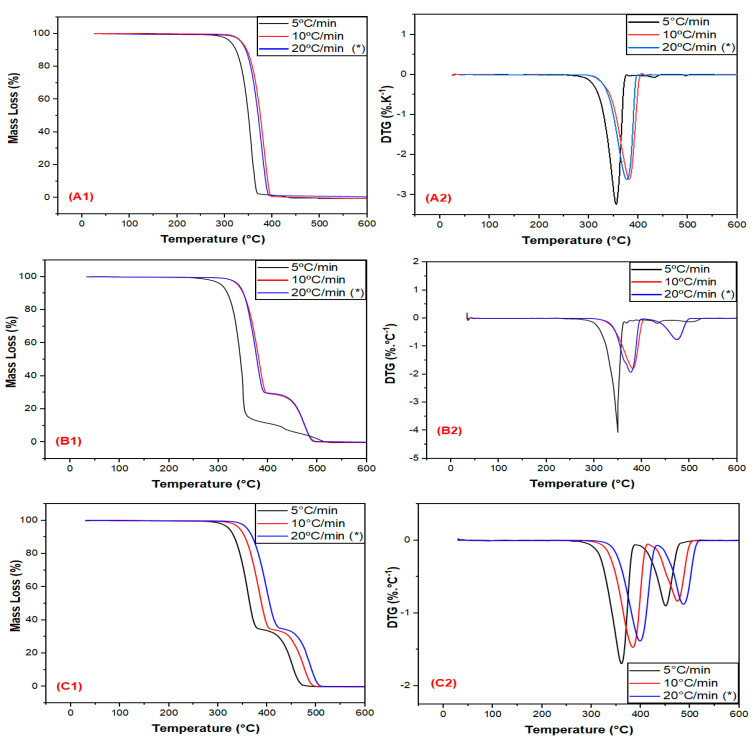
Mass loss and derivative curves vs. temperature of PLA (**A1**,**A2**), PLA30EE (**B1**,**B2**), and PLA30EE-C (**C1**,**C2**) at different heating rates. * Originally published: [17].

Figure 2 shows the mass loss (TG) and derivative (DTG) curves of PLA (A1, A2), PLA30EE (B1, B2) and PLA30EE-C (C1, C2) blends versus temperature at different heating rates.

The PLA/EE blends exhibited two thermal decomposition events and two DTG peaks (T_p1_ and T_p2_) regarding PLA (1st event) and EE (2nd event) degradation processes, as previously reported [17]. The presence of EE or EBAGMA did not change the PLA thermal decomposition onset temperature. These results agreed with the report by Hassan et al. [46] in their evaluation of the thermal degradation of PLA/ HDPE blends, in which these blends exhibited two thermal decomposition events associated with each polymer. The 1st event (relative to PLA) occurred between 325–425 °C and T_p, PLA_ was identified at 375.4 °C, while the HDPE decomposition (2nd event) occurred between 430–520 °C and T_p, HDPE_ was identified at 480.1 °C.

Table 6 shows the corresponding temperatures in which there are 5% (T_5%_) and 10% (T_10%_) of mass loss and the temperature (T_p_) where the degradation rate is maximum. The use of EBAGMA as a compatibilizer agent in the PLA30EE blend slightly shifted T_onset, PLA_ to higher temperatures, indicating that this compatibilizer agent could have increased the thermal stability of both PLA and EE. These results were similar to the investigation of Karimpour-Motlagh et al. [44] of the thermal stability of PLA/PP/cloisite composites compatibilized with EBAGMA. The presence of PP reduced T_onset, PLA_ in all heating rates. However, EBAGMA increased the T_onset_ of PLA/PP/cloisite composites, suggesting this CA could have improved PLA system thermal stability. On the other hand, Lu et al. [58] studied the thermal decomposition of PLA/HDPE blends compatibilized by EBAGMA. The T_onset, PLA_ increased regardless of HDPE%, and the %HDPE did not affect the T_p, HDPE_. However, adding 5%EBAGMA in PLA/HDPE 60/40 blend reduced T_5%_ from 343.2 to 314.1 °C and T_p, PLA_ from 367.2 to 354.5 °C.

### 3.2. PLA and PLA/EE Blends Kinetics of Thermal Degradation

#### 3.2.1. Estimative of Apparent Activation Energy (E_a_)

**Figure 3:**
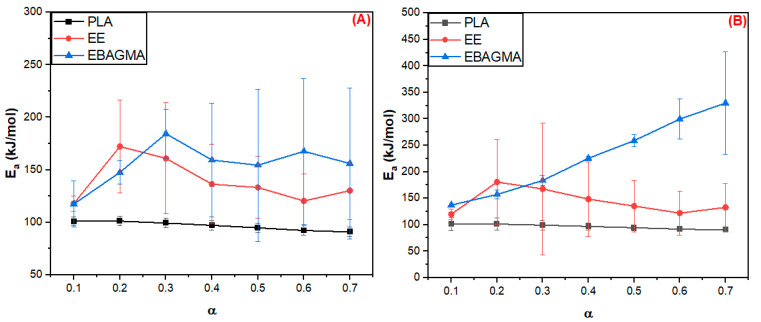
E_a_ vs. α estimated by FWO (**A**) and Vyazovkin (**B**) methods for the PLA, EE, and EBAGMA degradation reaction.

Figure 3 shows the activation energy (E_a_) estimated by FWO (Figure 3A) and Vyazovkin (Figure 3B) methods as a function of the conversion extension (α) for PLA, EE, and EBAGMA. E_a_ is associated with the susceptibility and reactivity of a chemical reaction and indicates the minimum energy required for breaking the molecular bonds [30]. The average value E_a_ $\left(\bar{E_{a}}\right)$ of PLA was approximately 96.6 kJ/mol by the FWO method and 96.4 kJ/mol by the Vyazovkin method, and E_a_ decreased slightly with the conversion rate α. Carrasco et al. [59] studied the PLA thermal degradation kinetics by the FWO method, reporting that PLA E_a_ varied accordingly to α, and $\bar{E_{a}}$ was estimated as 162 kJ/mol. According to Monika et al. [38], the PLA $\bar{E_{a}}$ was estimated as 158 kJ/mol by the FWO method. The divergence between the $\bar{E_{a}}$ value and the ones reported in the literature can be due to a difference in the molecular weight of PLA. In our work, PLA was previously processed in the mixer chamber before the TGA analysis, which could have led to a degradation of the fraction of higher molecular weight molecules.

The E_a_ average value $\left(\bar{E_{a}}\right)$ was approximately 138.8 (FWO method) and 143.8 kJ/mol (Vyazovkin method) for the ethylene elastomer EE and 155.3 (FWO method) and 227.4 kJ/mol (Vyazovkin method) for the acrylate compatibilizer EBAGMA, and E_a_ values were influenced by α in both polymers. Sinfronio et al. [60] research about low (LDPE) and high (HDPE) density polyethylene thermal degradation kinetic using the FWO method reported a similar tendency. The activation energy E_a_ varied with the conversion rate α, and the average $\bar{E_{a}}$ was 192.53 kJ/mol for LDPE and 202.46 kJ/mol for HDPE. On the other hand, Lyer et al. [61] employed the FWO method and reported an average $\bar{E_{a}}\;$ of 262.1 kJ/mol for the LDPE and 257.2 kJ/mol for the HDPE. The difference between the $\bar{E_{a}}$ values could be explained by the fact that EE is an amorphous thermoplastic elastomer with very low crystallinity, while LDPE and HDPE are thermoplastics with low and high crystallinity. In addition, the molecular weight of both polyethylenes is usually higher than the thermoplastic elastomers. The activation energy average $\bar{E_{a}}$ of EBAGMA increased drastically with the conversion rate α because of a possible transformation of the molecule’s chemical and physical structure at high temperatures, resulting in a more stable structure due to intra and intermolecular forces. Moreover, amorphous polymers like EBAGMA could have high entanglement density that would contribute to better thermal stability and higher E_a_ values. The curves E_a_ vs. α in Figure 3A and B showed the same profile and $\bar{E_{a}}$ values of the same order existing in good accordance with the models that suggest similar mechanisms of degradation for the individual polymers. Therefore, the EBAGMA presence in the PLA/EE mixture will improve the blend stability and the decomposition process [30].

**Figure 4:**
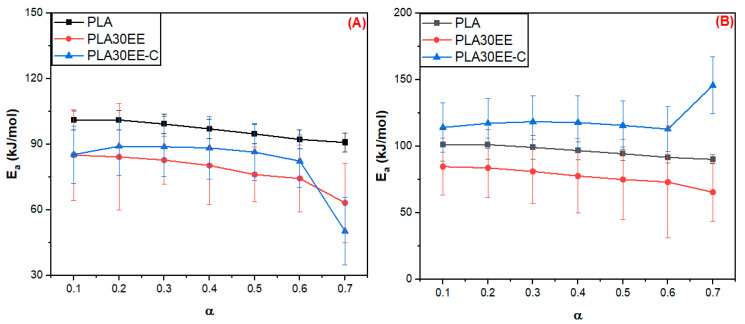
E_a_ estimated by FWO (**A**) and by Vyazovkin (**B**) methods as a function of α during the degradation reaction for PLA and PLA30EE blends.

Figure 4 shows the activation energy (E_a_) estimated by FWO (A) and Vyazovkin (B) methods as a function of the α for PLA and PLA/EE blends. The addition of 30 wt.% of EE in the PLA depressed the E_a_ of the PLA30EE blend compared to the neat PLA since the polymers are incompatible, as was already reported in the literature [17]. Harris et al.’s [62] study about PLA/HDPE identified that 10 wt.%HDPE has reduced the PLA onset thermal degradation, resulting in a faster decomposition process of PLA. On the other hand, the addition of 5 wt.% EBAGMA in the PLA30EE mixture improved the blend compatibility and thermal stability, increasing the activation energy required for starting the degradation process. The variation in the Ea values with conversion may be associated with a change in the reaction mechanism during thermal degradation.

Table 7 shows the activation energy E_a_ average values estimated by the FWO and Vyazovkin methods for the pure polymers (PLA, EE, EBAGMA) and PLA30EE blends. The presence of EBAGMA in PLA30EE blend increased the E_a_ value, which was an effect of the compatibilization promoted by EBAGMA on the physical and chemical intermolecular interaction between the PLA-EE molecules, producing a more stable system even at higher temperatures. A similar effect was reported by Reddy et al. [63] using 3 wt.% of the ethylene-propylene copolymer grafted with maleic anhydride (EP-g-MA) as a compatibilizer agent in PP/PLA blends, with the onset of degradation at higher temperatures due to a better stabilization of the mixture.

#### 3.2.2. Evaluation of Thermal Degradation Mechanism by the Criado Method

**Figure 5:**
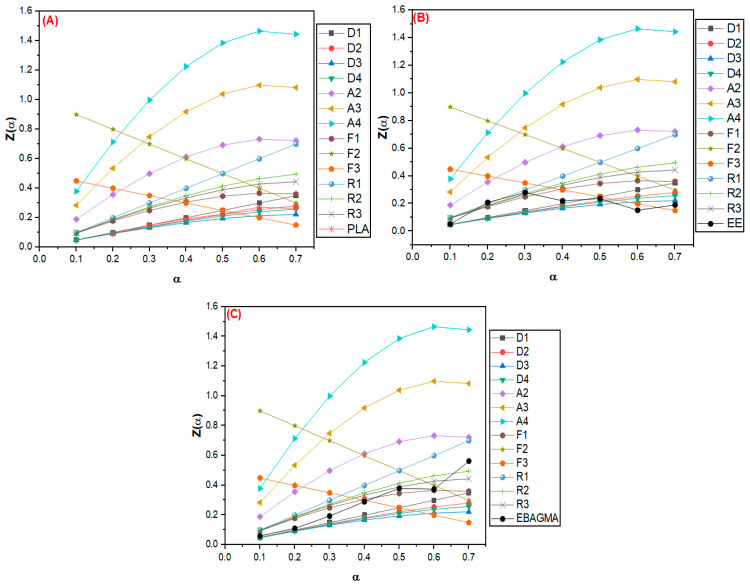
Master curves Z(α) vs. α of the PLA (**A**), EE (**B**), and EBAGMA (**C**) by Criado method.

The Criado method assessed the thermal degradation mechanism in a solid state of the PLA, EE, and EBAGMA. The activation energy E_a_ was estimated by the FWO method (as similarly done in [39]). Figure 5 shows the master curves Z(α) versus α and the experimental data of the PLA (A), EE (B), and EBAGMA (C).

The PLA degradation mechanism was related to the one-dimension diffusion mechanism (D1) and three-dimension diffusion (Ginstlinge-Brounshtein model) mechanism (D4). Considering that D1 and D4 are diffusional processes, it could be assumed that the reaction rate is higher than the reaction front propagation [64,65]. This result diverged from Alhulaybi et al. [31], Shao et al. [66], and Gharshallah et al.’s [67] findings, which suggested that PLA thermal degradation is more likely to happen as R2, R3, and F2 mechanisms, respectively. The divergence could be due to the scission of some PLA chains during processing by shear forces, reducing the average molecular weight, which would explain the lower degradation activation energy (E_a_) value found for PLA in this work (Table 7). As a result, the heat transfer to PLA may promote a degradation process based on a diffusion mechanism (D-type) instead of a phase boundary-controlled reaction (R-type).

EE showed a solid-state thermal degradation mainly influenced by the conversion rate (α) (Figure 5B). At the beginning of the thermal degradation process, the sample exhibited a diffusion mechanism, probably associated with the heat transfer to the elastomer. When the conversion rate was between 0.2 and 0.3, the phase boundary-controlled reactions (mainly R1 and R2 mechanisms) controlled the solid-state degradation process. At this stage, the EE thermal degradation could have generated thermal degradation products (such as unsaturated butane) based on a reaction mechanism controlled by the sample´s surface. This trend agreed with the findings reported by Aboulkas et al. [68] and Choudhary et al. [69] for LDPE and HDPE thermal degradation mechanisms described as R-type. When the degradation reaction reached α between 0.4 and 0.7, the reaction mechanism tended to random nucleation (F3), probably due to the random cleavage of the remaining polymer chain segments.

On the other hand, EBAGMA (Figure 5C) exhibited an F1 degradation mechanism characterized by random nucleation with one nucleus on the individual particle [39]. According to Poletto et al. [70] and Vyazovkin [71], in the F1 mechanism, there are no preferable sites in the reaction medium to start the thermal degradation reactions, and there are regions responsible for nucleate and evolution of these reactions. Considering that EBAGMA is a random terpolymer composed of ethylene segments, n-butyl acrylate, and glycidyl methacrylate, the reaction mechanism in the solid-phase reaction could occur based on the random scission of the polymer chain, and the degradation propagates based on the random nucleation.

**Figure 6:**
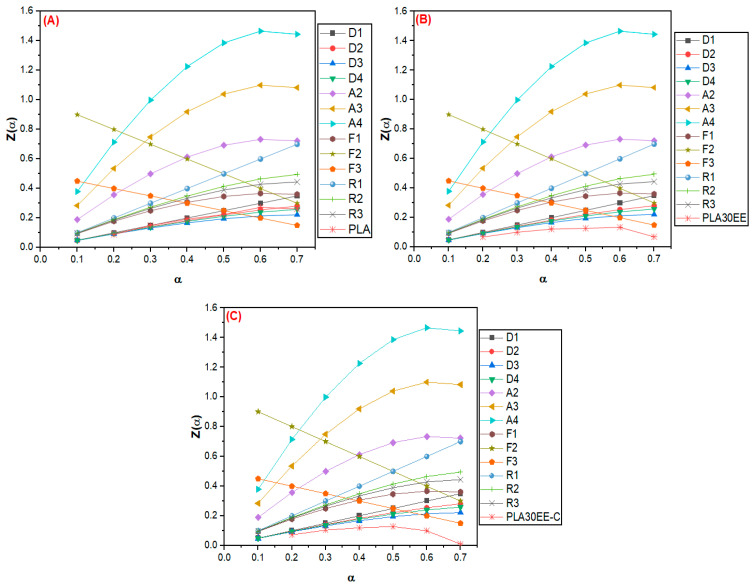
Master curves Z(α) vs. α of the PLA (**A**), PLA30EE (**B**), and PLA30EE-C (**C**) and experimental data from the Criado method.

The solid-state thermal degradations of PLA and PLA/EE blends were evaluated by the Criado method using the E_a_ estimated by the FWO method. Figure 6 shows the master curves and the results of the experimental data for the PLA (A) and blends PLA30EE (B) and PLA30EE-C (C).

The PLA30EE curve has shown a D-type degradation mechanism, the same exhibited by the individual polymers PLA and EE. The D-type mechanism suggested that the thermal degradation of PLA and EE did not affect each other, which could be explained by the different temperature range degradation of PLA and EE. The PLA onset thermal degradation process (see Figure 1 and Table 5) was earlier than EE, implying that EE is thermally more stable than PLA. The PLA30EE-C curve (Figure 6C) indicated this blend has also shown a D-type degradation mechanism (also exhibited by the individual PLA and EE, but not for EBAGMA). These results diverged from Karimpour-Motlagh et al. [44] research about the thermal degradation of PLA/PP blends with and without clay. PLA showed an F1-type thermal degradation, but it has converted into an F3-type in PLA/PP (75/25) blend, while the incorporation of clay into the polymer blend has changed the PLA thermal degradation mechanism from F3 to R2-type; however, when using EBAGMA in the reinforced polymer blend, the PLA mechanism has undergone a new modification (this time, from R2 to R3).

### 3.3. Thermodynamics Parameters of the PLA and the PLA/EE Blends

**Figure 7:**
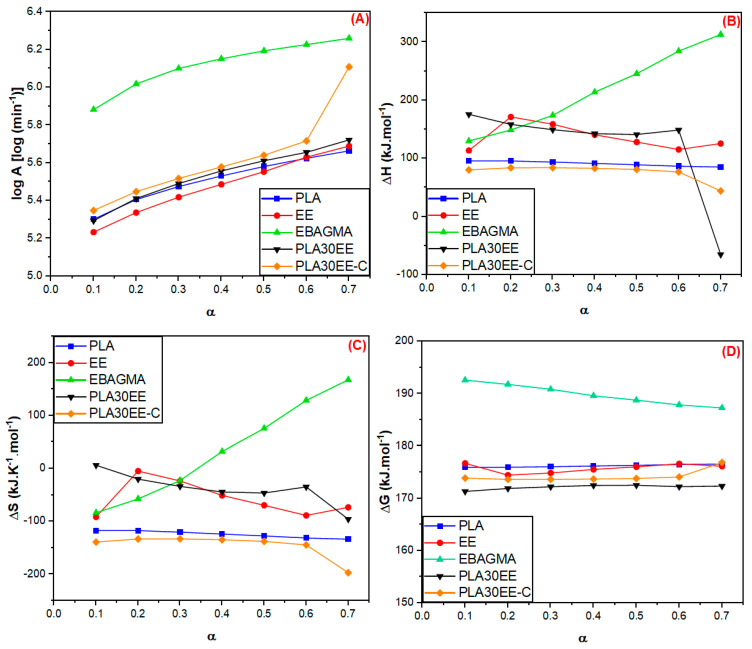
Frequency factor (**A**), molar changes in enthalpy (**B**), entropy (**C**), Gibbs free energy (**D**) as functions of extension of conversion (α) of the PLA, EE, EBAGMA, and PLA30EE blends estimated by FWO method.

The frequency factor (A), molar changes in enthalpy (ΔH), entropy (ΔS), and Gibbs free energy (ΔG) for the thermal decomposition reaction as functions of conversion of PLA, EE, EBAGMA, and PLA30EE blends are shown in Figure 7A–D, respectively). The estimative of each one of these thermodynamic parameters considered the activation energy (E_a_) values calculated according to the FWO method.

According to Choudhary et al. [69], higher frequency factors (A) values can indicate (i) high reactivity, (ii) higher barrier energy, and (iii) formation of a possible simpler complex during the reaction. For A ≥ 10^9^ min^−1^, a simpler complex is created during the reaction, while for A > 10^14^ min^−1^, the collision rate between the components of the system increases. According to Figure 7A, only EBAGMA presented A values higher than 10^14^ min^−1^, suggesting a higher activity of EBAGMA polymer segments during thermal degradation. EBAGMA possibly experienced higher reactivity than the other samples evaluated since the thermal degradation reaction was faster than the others. This result was corroborated by the highest value of ΔS of this polymer, which indicated a higher disorder degree of a system, and by the reaction mechanism in solid-phase reaction characterized by random scission of the polymer chain, as described previously.

The molar change in enthalpy (ΔH) (Figure 7B) can be interpreted as the total heat content of a system [72], and positive ΔH values indicative of an endothermic process during the thermal decomposition reaction [69], implying that the system requires heat absorption to progress the decomposition reaction. All samples presented positive ΔH values, which was expected once the energy absorption was needed to initiate the thermal degradation reaction. The PLA average ΔH value was 91.13 ± 4.25 kJ mol^−1^, a result lower than other values previously reported in the literature (114–160 kJ mol^−1^) [34,38]. This difference could be due to PLA thermal degradation during processing, reducing the length of some of the longer polymer chains and, consequently, E_a_ and ΔH values. On the other hand, EBAGMA presented the highest ΔH values, agreeing with the highest E_a_ (as seen in Table 7). The behavior of EBAGMA during thermal degradation suggested this terpolymer needed higher energy to initiate the thermal degradation process, and when it started, the reaction was faster and random.

The molar change in entropy (ΔS) (Figure 7C) indicates the randomness and disorder degree of a system [39,69]. Positive ΔS values suggest the disorder increased because the system changed, while negative ΔS values indicate the system became less disordered. According to Maia et al. [73], higher ΔS can be associated with the system reactivity, in which higher ΔS implies a faster reaction rate, decreasing the reaction time. PLA, EE, and both PLA30EE blends showed negative ΔS values, suggesting that the system was close to the thermodynamic equilibrium after the initiation of the thermal degradation reaction, agreeing with Palmay et al. [34] and Choudhary et al.’s [69] investigations. However, EBAGMA apparently exhibited a different behavior with the progression of conversion values. As the reaction progressed, the ΔS values changed from negative to the highest positive values. It is possible that the high reactivity of the reaction products increased the collisions between the molecules (as suggested by the A values) and promoted randomness in the degradation system, causing a higher system disorder and higher ΔS values.

The molar change in Gibbs free energy (ΔG) indicates how spontaneous a process is [69] under certain conditions (such as temperature, pressure, and composition). For positive ΔG, the thermal decomposition is non-spontaneous, requiring external agents to force the progress of this reaction. On the other hand, for negative ΔG, the thermal decomposition spontaneously occurs, and the activation energy to initiate the thermal decomposition decreases. All samples presented positive ΔG values (Figure 7D), and their thermal decomposition was non-spontaneous. EBAGMA presented the highest ΔG values (as commented before), suggesting this polymer needed a higher energy input to undergo thermal degradation. However, with the progress of the reaction, less energy was necessary for the thermal degradation continuity, which could be explained by probable nucleation and scission random of the polymer chain.

## 4. Conclusions

PLA, EE, EBAGMA, and PLA/EE blends were evaluated regarding their thermal stability, thermal degradation kinetics, and thermodynamic parameters. PLA/EE blends exhibited two thermal decomposition events, and the elastomer EE and the compatibilizer agent EBAGMA increased the thermal stability of PLA. The EBAGMA increased the activation energy (E_a_) average of the PLA/EE blend due to the compatibilization of the polymers compared to the blend without the compatibilizer agent. The solid-state thermal degradation evaluation using the Criado method revealed that PLA and its blends underwent degradation via a D-type mechanism, suggesting that thermal degradation was closely related to a diffusion mechanism. The increase in the collision rate between the polymer components during the thermal decomposition was probably the main factor for higher values of the thermodynamic parameters in the PLA/EE blend compared to the PLA and the compatibilized blend (PLA30EE-C).

## Figures and Tables

**Table 1:** Polymer’s main physical, rheological, and thermal data *, and suppliers.

Polymer	Density (g/cm^3^)	MFI (g/10 min)	T_g_ (°C)	T_m_ (°C)	Company
Poly (lactic acid) (PLA)	1.24	35 ^a^	60	170	NatureWorks
Ethylene elastomer (EE)	0.87	23 ^b^	−45	43	DuPont
Ethylene-butyl acrylate-glycidyl methacrylate (EBAGMA)	0.94	12 ^a^	−45	74	DuPont

* Condition: ^a^—190 °C/2.16 kg and ^b^—80 °C/2.16 kg.

**Table 2:** Nomenclature and compositions (wt.%) of the PLA/EE blends.

Sample	PLA/EE/EBAGMA (wt. %)
PLA	100/0/0
PLA30EE	70/30/0
PLA30EE-C	65/30/5

**Table 3:** Theoretical and experimental models for Z(α) type function.

Model	Z(α) Type Function
Theoretical	Z(α) = f(α)g(α)
Experimental	Z(α) = (dα/dT)(E_a_/R)(exp (E_a_/RT))P(x)

**Table 4:** Reaction thermodynamic parameters and respective equations.

Parameter	Equation
A	A = [β E_a_ exp (E_a_/RT_p_)]/RT_p_^2^
ΔH	ΔH = E_a_ − RT_α_
ΔG	ΔG = E_a_ + RT_p_ ln [(k_B_T_p_)/(hA)
ΔS	ΔS = (ΔH − ΔG)/T_p_

**Table 5:** TG and DTG parameters of PLA, EE, and EBAGMA.

Sample	Heating Rate (β) (°C/min)	T_5%_ (°C)	T_10%_ (°C)	T_p_ (°C)
PLA	5	312	324	358
	10	335	347	380
	20 *	334 *	365	378 *
EE	5	298	322	364
	10	317	337	375
	20	407	421	470
EBAGMA	5	377	396	456
	10	396	415	466
	20	405	422	470

* Originally published: [17].

**Table 6:** Temperatures with 5 and 10 wt.% mass loss, T_p_ according to heating rate of the PLA, and PLA/EE blends.

Sample	Heating Rate (β) (°C/min)	T_5%_ (°C)	T_10%_ (°C)	T_p,1_ (°C)	T_p,2_ (°C)
PLA	5	312	324	358	-
	10	335	347	380	-
	20	334 *	365	378 *	-
PLA30EE	5	331	341	365	459
	10	340	350	384	473
	20	341 *	364	377 *	474 *
PLA30EE-C	5	310	323	360	450
	10	344	355	385	475
	20	342 *	370	371 *	466 *

* Originally published: [17].

**Table 7:** Average activation energy (E_a_) of the PLA degradation, EE and EBAGMA, and PLA30EE blend estimated by FWO and Vyazovkin methods.

Sample	E_a_ (kJ/mol)	
	FWO Method	Vyazovkin Method
PLA	96.6 ± 4.4	96.4 ± 4.5
EE	138.8 ± 20.4	143.8 ± 23.0
EBAGMA	155.3 ± 20.4	227.4 ± 72.7
PLA30EE	78.0 ± 7.7	77.3 ± 6.8
PLA30EE-C	81.6 ± 14.0	120.4 ± 11.4

## Data Availability

The data presented in this study are available on request from the corresponding author.
